# Editorial: Investigating substance use disorders using neuroimaging-based brain connectivity

**DOI:** 10.3389/fpsyt.2022.992669

**Published:** 2022-08-08

**Authors:** Liangsuo Ma, Larry D. Keen, Marco Giuseppe Del Buono

**Affiliations:** ^1^Institute for Drug and Alcohol Studies, Virginia Commonwealth University, Richmond, VA, United States; ^2^Department of Psychiatry, Virginia Commonwealth University, Richmond, VA, United States; ^3^Wright Center, Virginia Commonwealth University, Richmond, VA, United States; ^4^Department of Psychology, Virginia State University, Richmond, VA, United States; ^5^Department of Cardiovascular and Pulmonary Sciences, Catholic University of the Sacred Heart, Rome, Italy

**Keywords:** substance use disorder, brain connectivity, fMRI, functional connectivity, resting state

Substance Use Disorders (SUDs) are chronic and relapsing with devastating personal, societal, and economic consequences. Given SUDs' wide-ranging impact, there is a pressing need in advancing our understanding of reliable risk factors, effective prevention strategies, and treatments for SUDs. Generally, neuroimaging-based brain connectivity (1) includes structural connectivity (anatomical links), functional connectivity (defined as statistical dependencies), or effective connectivity (reflecting causal interactions) among different regions within the brain. With fast and constantly evolving techniques, brain connectivity has gained immense potential in identifying neuronal correlates underlying risk factors, preventions, and treatments for SUDs. This Research Topic entitled, “*Investigating Substance Use Disorders using Neuroimaging-based Brain Connectivity*” aims to gather a diverse body of neuroimaging-based brain connectivity research studies to advance and/or consolidate our understanding of the neuronal correlates underlying the vulnerability, pathology, consequences, and treatment of the SUDs. Consistent with these aims, five articles were published under this Research Topic. All these five studies used resting state fMRI based functional connectivity but investigated five different substance use disorders.

Kuo et al. investigated heroin addiction by exploring the brain default mode network (DMN) using heroin-dependent individuals undergoing methadone treatment (MT, *n* = 11) and medication-free faith-based therapeutic community program (TC, *n* = 11). This study reported that compared with TC, MT had smaller functional connectivity between left and right inferior parietal lobe and between right inferior parietal lobe and posterior cingulate cortex and greater functional connectivity between medial prefrontal cortex and left inferior parietal lobe and between left inferior parietal lobe and posterior cingulate cortex. Further, the amplitude of low-frequency fluctuation (ALFF, a regional functional connectivity measure) (2) in left inferior parietal lobe was found to be positively associated with risk adjustment (decision-making performance) for all participants.

Suk et al. investigated Alcohol use disorder (AUD) by focusing one the salience network (SN), executive control network (ECN), and default mode network (DMN). Using functional MRI data and structural MRI data acquired from 22 AUD participants and 22 healthy controls, this study found that AUD individuals had greater functional connectivity within the DMN and SN networks (especially in terms of connectivity of the frontal areas and bilateral hippocampi) and lower functional connectivity in the ECN. In addition, there was significant volume reduction in these frontal areas and the hippocampus. The functional connectivity within both the frontal areas and bilateral hippocampi showed a negative correlation with gray matter volume of these areas in AUD patients. A strength of this study is the simultaneous examination of these networks using functional MRI and structural MRI.

Liu et al. investigated Betel quid dependence (BQD) using relatively larger sample sizes: 53 BQD individuals and 37 sex and age-matched healthy controls (HCs). Based on graph theoretical analysis (3) of a functional connectivity network with 90 nodes, this study reported that compared to HCs, BQ chewers had greater betweenness centrality (a nodal measure, reflecting the contribution rate of nodes in information exchange to other nodes) mainly in the right hemisphere and smaller betweenness centrality was found in the orbitofrontal area and temporal area which is associated with reward network, cognitive system and default mode network. In addition, BQD individuals presented the small-world (a global measure, reflecting the extent of the network between randomness, and order) topology but the normalized characteristic path length (λ, a global measure, representing the information transmission efficiency between the brain area and its neighboring) were greater than HCs. Furthermore, the area under curve (AUC) value of λ was found to be positively correlated with the duration of BQ chewing.

Based on previous studies indicating that overlapping pathophysiology may contribute to tobacco addiction and overweight, Zhang et al. used a mixed sample design to investigate neurobiological interaction mechanism between tobacco addiction and overweight status. This study reported a significant interaction effect between tobacco addiction and weight status in the functional connectivity in right superior frontal gyrus. In addition, the regional homogeneity (ReHo, a measure of local functional connectivity) (4) value in right superior frontal gyrus is positively associated with the tobacco addiction severity (pack-year). The findings of this study may have implication for treatments for individuals with comorbid tobacco addiction and overweight.

The above four studies used cross sectional design and therefore were not able to distinguish if the altered resting state networks were preexisting or due to substance use. This limitation did not apply to Camchong et al. however, as they employed longitudinal design to study cannabis use disorder. This study compared resting state functional connectivity changes in executive control and reward networks between 23 non-treatment seeking young adults with cannabis use disorder and 21 age-matched controls to determine group differences in the temporal trajectories of resting state functional connectivity across a 2-year span. Using seed-based functional connectivity method, this study reported sustained lower resting state functional connectivity of anterior cingulate cortex seeds with frontal and thalamic regions in the cannabis use disorder group *vs*. the age-matched controls. In addition, significant increases in functional connectivity between caudal anterior cingulate cortex and precentral and parietal regions over time were observed in the control group, but not in the cannabis use disorder group. The authors concluded that the altered executive control networks found in non-treatment seeking young adults with cannabis use disorder may impact regulatory control over substance use behavior.

Using different resting state functional connectivity techniques, these studies collectively showed that SUDs are associated with altered brain networks related to the prefrontal regions, an important part of the neurocircuitry underlying the neurobiology of SUDs (5). The findings from these studies advanced our understanding of the neuronal correlates that could underlie the vulnerability, pathology, consequences, and treatment of the SUDs. A major limitation of these studies was the small sample sizes. In addition, structural connectivity and effective connectivity analytical techniques were not used. Future studies with larger sample sizes using other brain connectivity techniques are warranted.

## Author contributions

LM drafted the original version of the editorial. LK and MD edited and approved the final version of the editorial. All authors contributed to the article and approved the submitted version.

## Conflict of interest

The authors declare that the research was conducted in the absence of any commercial or financial relationships that could be construed as a potential conflict of interest.

## Publisher's note

All claims expressed in this article are solely those of the authors and do not necessarily represent those of their affiliated organizations, or those of the publisher, the editors and the reviewers. Any product that may be evaluated in this article, or claim that may be made by its manufacturer, is not guaranteed or endorsed by the publisher.
